# Efficacy and organ protective effects of continuous renal replacement therapy in children with organic acidemia complicated by decompensated acidosis: a retrospective study in PICU

**DOI:** 10.1186/s13023-025-04169-2

**Published:** 2025-12-29

**Authors:** Lili Xing, Yueniu Zhu, Lianshu Han, LiLi Xu, Jiru Li, Jiayue Xu, Xiaodong Zhu

**Affiliations:** 1 Department of Pediatric Critical Care Medicine, Xinhua Hospital, Affiliated to Shanghai Jiaotong University School of Medicine, Shanghai, 200092 China; 2 Department of Pediatric Endocrinology and Genetic, Shanghai Institute for Pediatric Research, Xinhua Hospital Affiliated to Shanghai Jiao Tong University School of Medicine, Shanghai, 200092 China

**Keywords:** Organic acidemia, Continuous renal replacement therapy, Inherited metabolic disease, Decompensated metabolic acidosis

## Abstract

**Background:**

Metabolic decompensation is life-threatening in children with organic acidemia (OA). This study aims to evaluate the efficacy of continuous renal replacement therapy (CRRT) in treating patients with OA complicated by decompensated metabolic acidosis in pediatric intensive care units (PICU) and to explore its impact on organ function impairment associated with this condition.

**Methods:**

Data of children with OA developed decompensated metabolic acidosis admitted to PICU between 2013 and 2024 were collected and retrospectively analyzed. The patients were divided into two groups: the CRRT treatment group (admitted after 2019) and the conventional treatment group (admitted before 2019).

**Results:**

Forty-six patients were included in the study. After 24 hours of CRRT therapy, there was a significant decrease in levels of lactic acid, plasma ammonia, blood urea nitrogen, creatinine, troponin, myoglobin and neuron-specific enolase (NSE) compared to the conventional treatment group. After 48 hours of CRRT therapy, alanine aminotransferase and aspartate aminotransferase levels decreased in comparison with the conventional treatment group (*p* < 0.05). After 5 days of therapy, a significant decline in NSE persisted in the CRRT group compared to the conventional treatment group (*p* < 0.05). In the conventional group, organ injury biomarkers increased at 24 hours and gradually declined over time, whereas no elevation of these biomarkers was observed in the CRRT group. The duration of mechanical ventilation and the length of stay in the PICU were significantly shorter in the CRRT group (*p* < 0.05).

**Conclusions:**

In patient with OA complicated by decompensated metabolic acidosis, the treatment combined with CRRT can efficiently remove metabolites, thereby avoid neuron and the major organs from further injury. Application of CRRT can help these patients recover earlier from the metabolic crisis.

## Background

Organic acids are carboxylic acids produced during the metabolism of amino acids, fats, and sugars. Organic acidemia (OA) is typically caused by genetic metabolic disorders. An inherited deficiency in specific enzymes can lead to the accumulation of certain carboxylic acids and their metabolites, resulting in OA [1]. Severe cases of OA often result in multisystem damage or even death. Due to the absence of distinct symptoms and appropriate treatment, many children with OA are not admitted until a life-threatening metabolic crisis occurs [2]. Timely and effective interventions, particularly the removal of accumulating metabolites, can be lifesaving for these patients.

Continuous renal replacement therapy (CRRT), which includes hemodialysis and hemofiltration, is an extracorporeal circulation system that continuously removes metabolic waste and excess fluid from the blood. Initially employed for the treatment of renal failure, CRRT has become a crucial intervention for critically ill patients, aiding in kidney function support, toxin removal, and the correction of electrolyte and acid-base imbalances [3, 4]. Hemodialysis is recommended as the immediate management for children suffering from severe hyperammonemia due to urea cycle disorders [5]. However, there have been very few studies assessing the role of CRRT in managing decompensated acidosis in children with inherited metabolic disorders [6, 7]. Given that most organic acids are small-molecule, water-soluble compounds that are not protein-bound, they are apt to be cleared by dialysis. Since 2019, our hospital has implemented CRRT (including hemodialysis and hemofiltration) for children with decompensated metabolic acidosis due to OA as a first-line therapy. This therapy has shown potential as an adjunctive strategy to improve patient outcomes. The present study aims to evaluate the effects of the CRRT strategy in pediatric patients with OA developed decompensated metabolic acidosis by comparing laboratory values and clinical outcomes with those of patients receiving conventional treatment.

## Methods

### Patients

Pediatric patients with OA developed decompensated metabolic acidosis admitted to Xinhua Hospital affiliated with Shanghai Jiao Tong University School of Medicine from January 2013 to May 2024 were retrospectively analyzed. The present study identified patients with decompensated metabolic acidosis, defined by blood gas tests revealing blood pH < 7.25 and base excess of less than −3. The inclusion criteria were as follows: 1) OA had been previously diagnosed via tandem mass spectrometry (MS/MS) and gene analysis, 2) The age range was from 1 month to 18 years; 3) In the blood gas results, the pH value was less than 7.25 at admission. Prior to 2019, CRRT was solely employed as a salvage treatment for patients who had experienced failure of conventional therapy. With the continuous accumulation of clinical experience and supportive literature, our institutional protocol was revised in 2019. Since then, CRRT is recommended as the first-line treatment for decompensated metabolic acidosis due to OA, rather than as salvage therapy. The patients were stratified into two groups according to the treatment modality received: (1) the conventional treatment group, which included patients admitted between January 2013 and December 2018 and received standard treatment without CRRT; and (2) the CRRT treatment group, composed of patients admitted from January 2019 to May 2024 who received CRRT as an adjunct to conventional treatment upon admission.

All patients undergoing CRRT provided informed consent for central venous catheterization and blood purification treatment. This retrospective observation study received approval from the Ethics Committee of Xinhua Hospital Affiliated to Shanghai Jiao Tong University School of Medicine. The requirement for written informed consent for the study was waived by the committee, as all data were used retrospectively and de-identified (Approval No. XHEC-D-2024–197).

### Conventional treatment

The conventional treatment was defined as a comprehensive management including circulatory and ventilatory support, prevention of hypoglycemia and catabolism, correction of metabolic acidosis and electrolyte imbalance, and supplementation with cofactors and specialized diary.

During the initial 24–48 hours after admission, stop of feeding was implemented in some patients. To ensure sufficient energy intake and prevent catabolism, high-concentration dextrose solution and medium-chain triglycerides was administered intravenously. As clinical condition stabilized, the patients started to receive partial enteral nutrition and gradually advanced to full enteral feeding with a natural protein-restricted diets supplemented with disease-specific formula. Moreover, fluid therapy at 1.25–1.5 times the maintenance requirement was provided to enhance metabolic clearance. Metabolic acidosis and electrolyte imbalances were corrected through the intravenous infusion of electrolyte solutions and sodium bicarbonate. For patients with blood ammonia levels exceeding 100 μmol/L, intravenous arginine was administered promptly at a dosage of 200 mg/kg/d. Cofactors, including L-carnitine and vitamin B12, were supplemented to patients based on clinical indications [3–5]. Immediately upon admission, intramuscular administration of hydroxocobalamin (5–10 mg/d), was performed in the patients with methylmalonic acidemia, who had previously been identified as responsive to vitamin B12. In the conventional treatment group, hydroxocobalamin therapy was maintained for the first 5 days while intramuscular administration was temporarily withheld during the heparinized CRRT period in the CRRT treatment group.

In China, phenylbenzoate is accessible only in oral formulation, which is not suitable for critically ill patients, and carglumic acid is under Phase IV clinical trial in the Chinese population since 2024.

### CRRT treatment

The CRRT treatment was defined as treatment with CRRT in addition to conventional medical treatment. The CRRT was conducted within 12 hours after initial therapy for decompensate metabolic acidosis due to OA. Continuous Veno-Venous Hemodiafiltration (CVVHDF) was conducted utilizing the Prismaflex M60 membrane hemofilter, which equipped with an AN69 filter manufactured by Gambro Renal Products (Meyzieu, France) as part of a comprehensive continuous renal replacement therapy system (Gambro Prismaflex, Gambro Lundia Monitor Division, Lund, Sweden). Vascular access was achieved through central venous catheters ranging from 6F to 12F (GamCath; Gambro, Colombes, France), inserted into the right internal jugular vein or femoral vein based on the child’s age and weight. Unfractionated heparin served as the anticoagulant, provided with a loading dose of 30 IU/kg and a continuous maintenance dose of 20-30IU/kg/h. The parameters for hemodiafiltration were set as follows: a blood flow rate of 5 ml/kg/min, dialysis at 20–30 ml/kg/h, and posterior replacement fluid at 20 ml/kg/h. The replacement fluid was prepared using a modified Ports formula, incorporating electrolyte concentrations of sodium at 139 mmol/L, potassium at 3.7 mmol/L, bicarbonate at 34 mmol/L, calcium at 1.2 mmol/L, magnesium at 0.7 mmol/L, and glucose at 7 mmol/L.

### Measurements

Data were collected regarding the age, gender, Glasgow Coma Scale (GCS) scores, Pediatric Risk of Mortality (PRISM III) at admission, length of Pediatric Intensive Care Unit (PICU) stays, length of hospital stays, mechanical ventilation (MV) time, and mortality rates. Blood tests indicative of organ injury were performed at admission, as well as at 24 hours, 48 hours, and 5 days after the initiation of conventional treatment or CRRT treatment. The measured parameters included blood pH, Base excess (BE), glucose, lactic acid (Lac), plasma ammonia (PA), plasma homocysteine, hemoglobin (Hb), platelet count (Plt), plasma amylase, blood urea nitrogen (BUN), creatinine, alanine aminotransferase (ALT), aspartate aminotransferase (AST), troponin, myoglobin, and neuron-specific enolase (NSE).

### Statistical analysis

SPSS 26.0 (IBM Corp., Armonk, NY, USA) statistical software was employed to analyze the data. Normality tests were conducted to assess the distribution of all data. Measurement data that adhered to a normal distribution were presented as means ± standard deviation (x±s). Additionally, a t-test and one-way ANOVA were utilized for comparisons between the two groups. For data that did not conform to a normal distribution, median and interquartile ranges (IQR) were reported. The Mann-Whitney rank-sum test and Kruskal-Wallis test were applied for comparing non-normally distributed data. Enumeration data were expressed as counts or percentages (%), and the chi-square test was employed for analysis. A p-value of < 0.05 was considered statistically significant.

## Results

### Patient characteristics

**Fig. 1:**
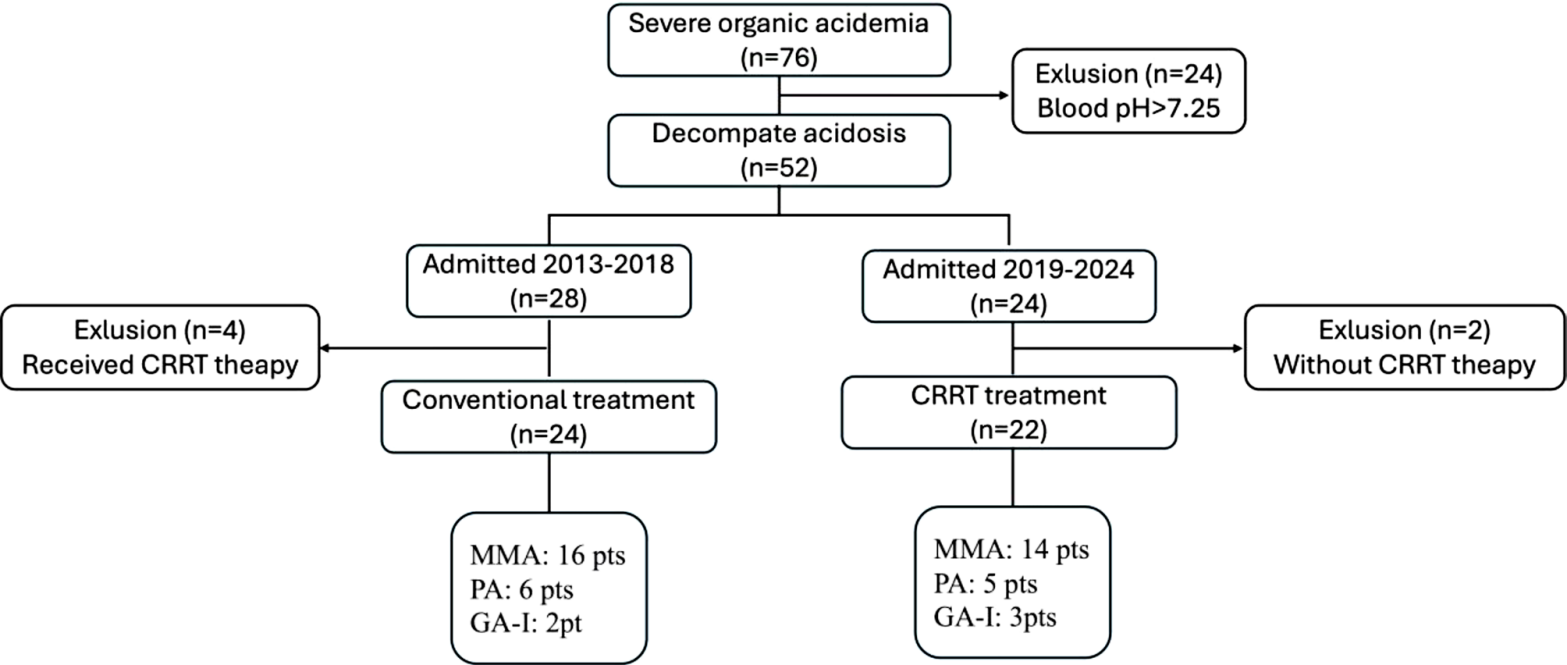
Flowchart of pediatric patients with severe organic acidemia

During the study period, 76 patients with OA were admitted to the PICU. Among them, 52 patients (68.4%) presented with decompensated metabolic acidosis. A total of 24 patients were assigned to the conventional treatment group, while 22 patients were included in the CRRT treatment group. The underlying inherited metabolic diseases of the included patients were methylmalonic acidemia (MMA) in 30 patients (65.2%), propionic acidemia (PA) in 11 patients (23.9%), and glutaric acidemia type 1 (GA-1) in 5 patients (10.9%, Fig. 1). Two gene subtypes, namely cblC-type and MUT-type, were identified in these patients with MMA. Patients with the cblC-type were responsiveness to vitamin B12, whereas all patients with the MUT-type in this study did not respond to vitamin B12. No significant differences in the metabolic diseases were observed between the groups (Table 1). Among these patients admitted between 2013 and 2018, four patients underwent CRRT after failure of conventional therapy were excluded in accordance with the inclusion criteria (Fig. 1). These criteria specifically required that CRRT therapy should be initiated immediately upon hospitalization, rather than being used as salvage therapy.

**Table 1:** Characteristics of the pediatric patients admitted with decompensate acidemia

	Conventional group (*n* = 24)	CRRT group (*n* = 22)	*p* value
Congenital disorder			
MMA, n (%)	16 (66.7%)	14 (63.7%)	0.829 ^b^
cblC-type	2 (8.3%)	2 (9.1%)	0.927 ^b^
MUT-type	14 (58.3%)	12 (54.5%)	0.796 ^b^
PA, n (%)	6 (25%)	5 (22.7%)	0.857 ^b^
GA-1, n (%)	2 (8.3%)	3 (13.6%)	0.564 ^b^
Age (months), mean±SD	33.5 ± 11.3	42.7±13.5	0.293^c^
Male, n (%)	16 (66.7%)	14 (63.6%)	0.830^b^
GCS, medium (IQR)	12 (11, 13)	12 (10, 13)	0.878^a^
PRISMIII, medium (IQR)	13 (12, 15.8)	15 (12.25, 17.8)	0.182^a^
pH, medium (IQR)	7.18 (7.14, 7.21)	7.21 (7.11, 7.25)	0.497^a^
BE, medium (IQR)	−19.3 (−22.6, −15.4)	−19.0 (−21.6, −16.6)	0.665^a^
Glucose(mmol/L), medium (IQR)	5.7 (4.3, 6.9)	7.8 (5.6, 10.0)	0.051^a^
Lac(mmol/L), mean±SD	9.6 ± 7.9	13.2 ± 9.1	0.257^c^
PA (umol/L), medium (IQR)	51.0 (36.3,122.0)	63.5 (31.3, 124.0)	0.821^a^
Plasma homocysteine (umol/L), mean±SD	26.0 ± 13.1	34.6 ± 25.0	0.262^c^
Hb (g/L), mean±SD	82.3± 11.7	86.9 ±9.6	0.186^c^
Plt (10^9/L), mean±SD	274.8± 119.2	250.9± 101.2	0.495^c^
Plasma amylase(u/L), medium (IQR)	45 (30.5, 55.0)	44(30.3,57.3)	0.547^a^
BUN (mmol/L), medium (IQR)	5.9 (3, 13.2)	4 (3, 9.3)	0.649^a^
Creatinine(umol/L), medium (IQR)	37.6 (30.0, 66.5)	47.9 (26.1, 101.0)	0.638^a^
ALT(u/L), medium (IQR)	38.0 (27.3, 124.3)	81.0 (27.8, 207.7)	0.407^a^
AST(u/L) medium (IQR)	67.0 (46.3, 204.8)	106.6 (48.8, 317.0)	0.497^a^
Troponin(ng/mL), medium (IQR)	0.018 (0.009, 0.03)	0.074 (0.011, 0.58)	0.090^a^
Myoglobin(ng/mL), medium (IQR)	33.0 (22.7, 147.7)	62.1 (26.9, 487.5)	0.187a
NSE (ng/mL), mean±SD	53.8 ± 14.8	61.0 ± 13.0	0.166c

a: Mann - Whitney rank-sum test, b: chi-square test, c: t-test

There were no significant differences between the two groups regarding age, gender, GCS score, and PRISM III scores at admission (*p* > 0.05, Table 1). Similarly, no significant differences were observed in organ function tests, including blood pH, BE, glucose, Lac, PA, plasma homocysteine, Hb, Plt, plasma amylase, BUN, creatinine, ALT, AST, troponin, myoglobin, and NSE at admission (*p* > 0.05, Table 1).

### Effect of CRRT treatment 24 hours after therapies

Twenty-four hours after commencing CRRT therapy, the CRRT group exhibited a significant reduction in metabolite levels, including Lac and PA, compared to the conventional treatment group (*p* < 0.05). Furthermore, biomarkers indicating renal, cardiac, and neurological injury, such as creatinine, BUN, troponin, and NSE, showed marked decreases in the CRRT treatment group compared with conventional group (*p* < 0.05). However, no significant differences were noted between the groups in the correction of acidosis or in the biomarkers of hematological system, pancreatic, hepatic and muscular injury, including pH, BE, glucose, plasma homocysteine, Hb, Plt, plasma amylase, ALT, AST, and myoglobin (*p* > 0.05, Table 2).

**Table 2:** Effect of CRRT treatment 24 hours, 48 hours and 5 days after therapies

	24 hours		48 hours		5 days	
	Conventional group (*n* = 24)	CRRT group (*n* = 22)	Conventional group (*n* = 23)	CRRT group (*n* = 22)	Conventional group (*n* = 20)	CRRT group (*n* = 20)
pH, medium (IQR)	7.38 (7.27,7.44)	7.35 (7.26,7.42)	7.31 (7.24, 7.43）	7.37 (7.30, 7.46)	7.41 (7.32, 7.43)	7.41 (7.37, 7.44)
BE, mean±SD	−5.8 ± 7.3	−5.2 ± 6.8	−5.5 ± 8.9	−2.6 ± 5.8	−4.3 ± 7.5	−1.5 ± 4.1
Glucose(mmol/L), medium (IQR)	6.9 (5.6, 9.6)	8.0 (7.2, 10.1)	7.1 (6.2, 9.0)	7.7 (6.6, 8.9)	5.1 (4.1, 6.2)	6.6 (5.6, 8.2)
Lac(mmol/L), mean±SD/medium	7.8 ± 3.9	5.4 ± 2.1 *	6.8 ± 4.1	4.8 ± 2.6 *	2.8 (2.0, 6.5)	3.8 (1.8, 7.8)
PA (umol/L), medium (IQR)	52.0 (23.0, 98.0)	25.0 (11.5, 57.8) *	56.0 (28.0, 102.0)	23.0 (8.0, 53.8) *	38.0 (16.3, 65.5)	26.0 (16.0, 32.5)
Plasma homocysteine (umol/L), medium (IQR)	21.4 (7.4, 39.7)	14.4 (7.4, 24.9)	24 (17.8, 28.2)	9.15 (6.1, 20.3) *	20.6 (7.3, 30.9)	5.3 (3.9, 8.2) *
Hb (g/L), Medium (IQR)/mean±SD	82.1 (73.0, 87.5)	80.3 (75.3, 87.3)	80.6 ± 11.8	85.1 ± 10.7c	93.5 ± 12.1	97.9± 12.8
Plt(10^9/L), mean±SD	265.4 ± 118.1	213.5 ± 85.9	203.6 ± 107.4	163.7 ± 79.6	237.3± 107.7	199.7± 104.5
Plasma amylase (u/L), medium (IQR)	63.2 (44.0, 94.5)	52.2 (42.0, 64.5)	72.5 (45.8, 161.8)	53.5 (38.3, 79.8)	43.2 (36.3, 92.1)	37.0 (31.8, 72.5)
BUN (mmol/L), medium (IQR)	7 (4, 10.2)	2 (1.3, 3.9) *	6.8 (3.8,9.4)	3 (1.8, 4.1) *	3.8 (1.9, 6.3)	4.5 (2.8, 7.4)
Creatinine (umol/L), medium (IQR)	45.3 (25, 55.9)	24.5 (17.8, 37.8) *	31.2 (24, 59.5)	27.6 (17.3, 46.3)	27.4 (13.8, 41.1)	23.0 (19.9, 56.6)
ALT(u/L), medium (IQR)	94.5 (44.0, 187.0)	76.8 (35.3, 267.5)	107.0 (32.0, 195.0)	61.5 (33.8, 96.0) *	82.5 (27.5, 133.8)	60.0 (30.0, 102.5)
AST(u/L) medium (IQR)	123.0 (59.0, 247.0)	94.0 (48.6, 391.0)	137 (39.5, 333)	83 (33.5, 176) *	102.9 (36.5, 327.5)	65 (44.9, 140.5)
Troponin(ng/mL), medium (IQR)	0.4 (0.2, 2.2)	0.07 (0.02,0.08) *	0.2 (0.01, 0.6)	0.02 (0.007, 0.1) *	0.01 (0.002, 0.06)	0.02 (0.002, 0.2)
Myoglobin(ng/mL), medium (IQR)	37.0 (18.2, 87.0)	128.7 (23.6, 995.3)	27.9 (15.5, 92.0)	61.4 (22.8, 196.0)	21.4 (8.6, 42.4)	35.7 (13.0, 98.9)
NSE (ng/mL), mean±SD，medium	62.9 ± 17.1	50.6 ± 11.1 *	50.5 ± 20.9	40.4 ± 9 0.0	32.1 (23.6, 38.3)	19.0 (14.0, 27.0) *

Data adhered to a normal distribution were presented as means ± standard deviation (x ± s). A t-test was utilized for comparisons between the two groups at the same timepoint of the therapies. Data with non-normalized distribution, median and interquartile ranges (IQR) were reported. The Mann-Whitney rank-sum test and Kruskal-Wallis test were applied for comparing the difference between the two groups at the same timepoint of the therapies. ‘*’ represent the significant difference between the groups at the same timepoint of therapies.

### Effect of CRRT treatment 48 hours after therapies

As shown in Table 2, after 48 hours of treatment, further improvements in metabolite levels, such as Lac and PA, were observed in the CRRT group. At this time point, biomarkers indicating renal and cardiac injury significantly decreased in the CRRT group, while hepatic injury biomarkers also showed a reduction compared to the conventional treatment group (Table 2, *p* < 0.05). However, there were no significant differences between the groups in acidosis correction or biomarkers of hematological system, pancreatic, neurological and muscular injury (Table 2, *p* > 0.05). Notably, one child in the conventional treatment group passed away 32 hours after the initial therapy.

### Effect of CRRT treatment 5 days after therapy

As shown in Table 2, after 5 days of CRRT therapy, the CRRT group exhibited a significant improvement in neurological injury biomarker compared to the conventional treatment group (Table 2, *p* < 0.05). However, no significant differences were observed in the metabolites and biomarkers of hematological system, pancreatic, renal, hepatic, and cardiac injury between the two groups (Table 2, *p* > 0.05). By the fifth day following the initial treatment, four children in the conventional treatment group and two children in the CRRT treatment group had died. In the conventional treatment group, one patient passed away due to refractory metabolic acidosis. This persistent acidosis triggered circulatory collapse and coagulopathy, eventually leading to the patient’s death. Regarding the other three patients in the conventional treatment group and the two patients in the CRRT treatment group, the primary cause of death was progressive infection, which subsequently led to multisystem organ failure (MOF).

### Prognoses and adverse effects of CRRT treatment

The median duration of CRRT treatment was 55 hours. The CRRT group experienced 10 hours less mechanical ventilation compared to the conventional treatment group (Table 3, *p* = 0.031). Additionally, the PICU stay for the CRRT group was nearly 3 days shorter than that of the conventional group (Table 3, *p* = 0.039), while there was no significant difference in overall hospital stay duration between the two groups (Table 3, *p* = 0.492). Mortality up to discharge was 18.19% (4/22) in the CRRT group and 20.83% (5/24) in the conventional group, with no statistically significant difference between the two groups (*p* = 0.821). Importantly, no serious adverse effects were noted, such as severe bleeding requiring transfusion and circulatory instability associated with extracorporeal circulation.

**Table 3:** Prognosis of the two groups

	Conventional group (*n* = 24)	CRRT group (*n* = 22)	p value
Duration of MV time (hour), medium (IQR)	25.5(6.6, 68.8)	14.3(0,73.9)	0.031a
PICU stays duration(day), medium (IQR)	12.3(7.6, 18.7)	8.5(4.9,12.1)	0.039a
Hospital stays duration(day), medium (IQR)	15.2(6.9, 20.8)	12.9(6.5,18.6)	0.492a
Mortality rate %(n)	20.83 (5)	18.19 (4)	0.821b

a: Mann - Whitney rank-sum test, b: chi-square test

### Effect of CRRT on organ injury

**Fig. 2:**
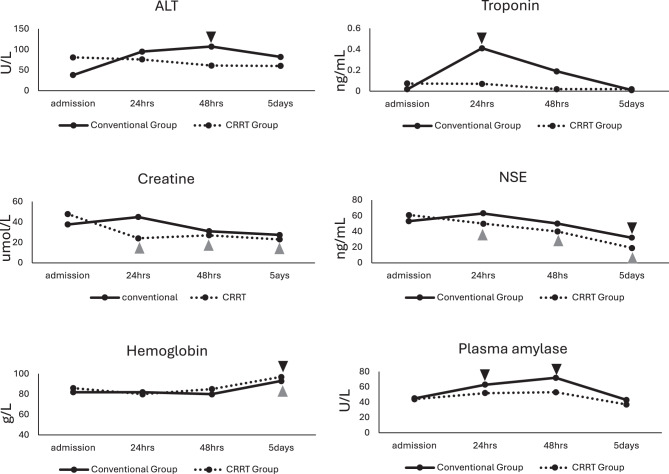
Line chart of organ injury biomarkers of the groups  represents significant difference compared with the value measured at admission in the coventional treatment group (*p* < 0.05, Kruskal-Wallis test).  represents significant difference compared with the value measured at admission in the CRRT treatment group (*p* < 0.05, Kruskal-Wallis test)

On the fifth day after therapy, the hemoglobin levels in both treatment strategy groups exhibited a significant increase when compared with the levels upon admission. In the conventional treatment group significant increases in hepatic injury biomarkers (ALT) and pancreases injury biomarkers (plasma amylase) were observed 48 hours after the initial treatment compared to those values at admission, while these biomarkers remained stable in the CRRT group throughout the therapy. The cardiac injury biomarker (troponin) in the conventional therapy group rose notably at 24 hours after admission, whereas no changes in troponin levels were detected in the CRRT group compared to the value of admission. The nervous system injury biomarker (NSE) was elevated in both groups at admission, with levels in the conventional group remaining high until the fifth day post-therapy. In contrast, NSE levels in the CRRT group decreased significantly throughout the treatment period. Forty-eight hours after the initial therapy, the pancreatic injury biomarker (plasma amylase) in the conventional treatment group showed an obvious elevation compared to the level at admission. Conversely, no significant increase was detected in the CRRT group during the entire course of the therapy. The renal injury biomarker (creatinine) was in the normal range for both groups, while a further reduction in creatinine levels observed in the CRRT group following therapy. (Fig. 2)

## Discussions

Organic acidemia (OA) is primarily caused by inherited metabolic disorders. Patients typically exhibit a blockage in the abnormal catabolism of amino acids and lipids, resulting from deficient activity of specific enzymes. The accumulation of these metabolites can lead to severe acidosis and, more critically, toxic organic acids can cause multiorgan damage [2, 8, 9]. In China, tandem mass spectrometry (MS/MS) is employed for newborn screening (NBS) of inborn errors of metabolism in approximately 60–70% of newborns. Among the patients identified via NBS, 26.8% exhibited clinical symptoms during the neonatal period. In the clinically diagnosed patients, the median age at diagnosis was 2.5 months [10]. MMA, PA and GA-I are among the most common forms of OA [1, 11–13]. Currently, hemodialysis is not routinely performed in neonates at the present hospital.

Metabolic decompensation can occur in these patients following an acute infection, vomiting, surgery, or trauma. Due to the lack of distinct features during the decompensation process, these patients are often not identified until they develop multiorgan dysfunction and life-threatening conditions. Such acute metabolic crises can lead to mortality rates as high as 40% in this population [14, 15]. Immediate management of these metabolic emergencies is crucial for affected children. Conventional interventions include ventilatory support, fluid resuscitation, correction of acidosis, provision of cofactors, and removal of toxic metabolites [16, 17]. For children experiencing metabolic decompensation, aggressive treatment may be lifesaving and can help reduce neurological sequelae. Compared to peritoneal dialysis, temporary hemodialysis or hemofiltration is frequently advocated for a more efficient correction of acidosis. [18, 19].

CRRT is an extracorporeal circulation system that can be performed using hemodialysis and hemofiltration. This procedure effectively removes excess fluid, inflammatory mediators, and metabolic waste from the blood, while also correcting metabolic derangements. Consequently, CRRT is widely utilized in the treatment of renal failure, severe electrolyte imbalances, and systemic inflammatory responses [20–22]. In the context of inherited metabolic diseases, hemodialysis is recommended as a management strategy for children experiencing severe hyperammonemia due to urea cycle disorders [5]. Research has demonstrated that CRRT is effective in treating hyperammonemia and propionic acidemia [23]. However, there is very limited research on the application of CRRT for managing decompensated metabolic acidosis in children with organic acidemia.

The AN69 equipment utilized in this context includes filtration capable of removing small and middle molecules, specifically those under 500 Da [24]. In situations of life-threatening acute metabolic crises, it is crucial to rapidly decrease the concentrations of plasma toxic metabolites, such as organic acids and ammonia. The most prevalent organic acidemias encountered include methylmalonic acidemia, propionic acidemia, and glutaric acidemia, with molecular weights ranging from 74 Da to 132 Da. Notably, these acids are water-soluble and exhibit low protein binding. Therefore, it is theoretically feasible to eliminate the toxic metabolites in children with organic acidemia through CRRT treatment. Our previous clinical practice found the effectiveness of CRRT treatment in part of the patients experiencing metabolic crises. Since 2019, we have recommended CRRT—which encompasses hemodialysis and hemofiltration therapy—as an adjunctive treatment for patients with MMA, PA, and GA-I who present with decompensated metabolic acidosis (blood pH < 7.25).

All patients included in the study presented with decompensated metabolic acidosis. Within 24 to 48 hours of the combined treatments, metabolites such as lactic acid, plasma ammonia, and urea nitrogen were significantly reduced in the CRRT group compared to the conventional therapy group. These results demonstrate that CRRT can efficiently remove toxic metabolites in OA patients complicated by decompensated metabolic acidosis, as predicted by the underlying mechanism. There was no difference in blood pH and base excess between the groups, as patients in the conventional therapy group typically received repeated doses of sodium bicarbonate to correct metabolic acidosis. This therapy can also increase blood pH and decrease base excess.

In this study, we found that CRRT significantly reduced organ injury. The levels of NSE and troponin, which indicate neuronal and cardiac injury, were markedly lower in the CRRT group compared to the conventional group at 24 hours post-therapy, and these trends persisted into the later stages of treatment. Additionally, creatinine and ALT levels were also reduced in the CRRT group at both 24 and 48 hours when contrasted with conventional therapy. Further analysis (Fig. 2) revealed that all organ injury biomarkers increased at 24 hours in the conventional group and gradually declined over time. In contrast, no elevation of organ injury biomarkers was observed in the CRRT group. NSE, an enzyme that released after neuronal damage, has been investigated as a biomarker for brain injury, including conditions such as central nervous system infections, hypoxic-ischemic encephalopathy and traumatic brain injury [25, 26]. The levels of NSE were found elevated at admission in both groups. In the conventional group, NSE levels slightly increased at 24 hours and did not decline until five days after treatment, whereas significant reductions in NSE were evident at all time points in the CRRT group. These findings suggest that CRRT treatment effectively mitigates major organ injury and alleviates neurological damage. The observed protective effects may be attributed to the rapid elimination of toxic metabolites in the CRRT treatment group.

We found that the duration of mechanical ventilation and the length of PICU stay was significantly shorter in the CRRT treatment group compared to the conventional treatment group. These findings indicate that patients in the CRRT group appear to recover more quickly from critical conditions than those in the conventional group. This advantage may be attributed to the effective clearance of toxic metabolites and the prevention of further organ injury.

There are several limitations that merit discussion. First, OA such as MMA, PA, and GA-1 are rare disease with low incidence worldwide. And this is a single-center retrospective study. The sample size eligible for the study was small. Second, during the emergency treatment in the PICU, we did not measure the levels of the specific toxic metabolites (e.g. propionylcarnitine(C3), acetylcarnitine (C2), methylmalonic acid, 2-methylcitric acid, and 3-hydroxypropionic acid) in real time to demonstrate the effect of CRRT on organic acid removal. This is because the MS/MS and GC-MS test are not performed in the hospital’s central laboratory. The levels of these metabolites were monitored after the acute episode of metabolic decompensation. Thirdly, as this is a retrospective study and the group assignment was based on chronological order, the presence of inevitable biases is acknowledged. For instance, patients failed in conventional treatment and received CRRT treatment at the end stage of the metabolic crisis were not included in the study. Given the limited research on CRRT treatment for OA, our findings may not fully represent the general practices in PICUs. Nonetheless, this study serves as a foundational step towards enhancing and optimizing CRRT treatment for metabolic decompensation in patients with OA. Prospective research or additional research involving larger cohorts would be beneficial to validate these preliminary findings.

## Conclusions

In conclusion, CRRT treatment can remove metabolite efficient in the early phase of therapy in patient with OA complicated by decompensate metabolic acidosis. The treatment combined with CRRT therapy can avoid neuron and the major organs from further injury. Application of CRRT treatment as an additional strategy can help these patients recover earlier from the metabolic crisis.

## Data Availability

The data that support the findings of this study are available from Xinhua Hospital affiliated to Shanghai Jiaotong University School of Medicine, but restrictions apply to the availability of these data, which were used under license for the current study, and so are not publicly available. Data are however available from the authors upon reasonable request and with the permission of Xinhua Hospital.

## Abbreviations

OA: organic acidemia
CRRT: continuous renal replacement therapy
PICU: pediatric intensive care units
NSE: neuron-specific enolase
MV: mechanical ventilation
BE: base excess
Lac: lactic acid
PA: plasma ammonia
Hb: hemoglobin
Plt: platelet count
BUN: blood urea nitrogen
ALT: alanine aminotransferase
AST: aspartate aminotransferase
IQR: interquartile ranges
MMA: methylmalonic acidemia
PA: propionic acidemia
GA-1: glutaric acidemia type 1
MS/MS: tandem mass spectrometry
NBS: newborn screening
